# Cytokine-augmented risk stratification for six-month incident nephritis in children with IgA vasculitis

**DOI:** 10.3389/fped.2026.1902940

**Published:** 2026-08-26

**Authors:** Fan Liu, Yang Pan, Yan Ding, Dan Wang

**Affiliations:** 1 Department of Rheumatology and Immunology, Wuhan Children’s Hospital (Wuhan Maternal and Child Healthcare Hospital), Tongji Medical College, Huazhong University of Science & Technology, Wuhan, Hubei, China; 2 Department of Laboratory Medicine, Wuhan Children’s Hospital (Wuhan Maternal and Child Healthcare Hospital), Tongji Medical College, Huazhong University of Science & Technology, Wuhan, Hubei, China; 3 Department of Gastroenterology, Wuhan Children’s Hospital (Wuhan Maternal and Child Healthcare Hospital), Tongji Medical College, Huazhong University of Science & Technology, Wuhan, Hubei, China

**Keywords:** children, cytokines, IgA vasculitis, nephritis, risk stratification

## Abstract

**Background:**

Children with IgA vasculitis (IgAV) may develop nephritis after initially presenting without kidney involvement. We evaluated whether a cytokine augmentation score (CAS) was associated with improved six-month incident nephritis risk stratification beyond clinical variables and routine inflammatory markers.

**Methods:**

This single-center cohort screened children younger than 18 years with IgAV from January 2023 to June 2024 and used standardized six-month renal outcome ascertainment. Children meeting accepted IgAV classification criteria, with no nephritis during the baseline decision window, complete baseline biomarker data, and six-month renal outcome ascertainment formed the cohort. A routine inflammatory index score (RIIS) summarized *z*-transformed natural-log C-reactive protein-to-albumin ratio (CAR), neutrophil-to-lymphocyte ratio (NLR), and systemic immune-inflammation index (SII); CAS summarized *z*-transformed natural-log interleukin (IL)-6, tumor necrosis factor alpha (TNF-α), IL-17A, IL-18, and IL-23. Multivariable logistic regression compared a routine clinical plus RIIS model with a cytokine-augmented model; the area under the receiver operating characteristic curve (AUC), Brier score, calibration, bootstrap optimism correction, decision-curve net benefit, and sensitivity analyses were evaluated.

**Results:**

Among 447 screened children, 337 were analyzed; 91 (27.0%) developed incident nephritis. CAS was independently associated with nephritis after adjustment for age, sex, symptom-to-blood-draw interval, gastrointestinal involvement, systolic blood-pressure percentile, subthreshold urinary warning signs, and RIIS (adjusted odds ratio per standard deviation (SD), 2.97; 95% confidence interval (CI), 2.01–4.39; *p* < 0.001). Adding CAS improved the AUC from 0.724 to 0.801 (Δ0.077; 95% CI, 0.035–0.124; *p* = 0.001), reduced the Brier score from 0.168 to 0.149, and yielded an optimism-corrected AUC of 0.782.

**Conclusions:**

In this single-center derivation cohort, CAS improved internally evaluated discrimination, Brier score, and risk separation beyond clinical variables and RIIS. The model remains investigational pending external validation, assay standardization, and implementation testing.

## Introduction

IgA vasculitis (IgAV), historically termed Henoch-Schönlein purpura, is the most frequent systemic small-vessel vasculitis of childhood and typically involves the skin, joints, gastrointestinal tract, and kidneys (1). Kidney involvement is the principal determinant of long-term morbidity because hematuria, proteinuria, hypertension, or decreased kidney function may emerge after an initially reassuring renal assessment (2). Contemporary pediatric reviews emphasize that IgAV is common but heterogeneous, with clinical phenotypes ranging from self-limited purpura to nephritis requiring prolonged surveillance (3). Classification is usually operationalized with the European League Against Rheumatism/Paediatric Rheumatology International Trials Organisation/Paediatric Rheumatology European Society (EULAR/PRINTO/PRES) criteria, which require lower-limb-predominant purpura or petechiae plus at least one supportive feature such as abdominal pain, arthritis or arthralgia, renal involvement, or IgA-predominant histopathology (4).

National recommendations advise frequent urinalysis over a 6-month period and blood-pressure assessment in children and young people with IgAV (5), while International Pediatric Nephrology Association (IPNA) recommendations define IgAV nephritis by clinical renal abnormalities including hematuria, proteinuria, hypertension, and reduced kidney function (2). The biological basis for delayed nephritis is plausible because IgAV pathogenesis involves IgA-containing immune complexes, endothelial inflammation, complement activation, neutrophil recruitment, and T-helper immune dysregulation (6, 7). However, current care still relies primarily on serial urine and blood-pressure checks because no baseline biomarker panel has been sufficiently validated to replace surveillance. A risk-stratification tool that identifies low-, intermediate-, and high-risk children could support follow-up intensity and family counseling without weakening the obligation to monitor all children.

Routine inflammatory markers are attractive because they are objective, inexpensive, and available at the index encounter. The C-reactive protein-to-albumin ratio (CAR), neutrophil-to-lymphocyte ratio (NLR), and systemic immune-inflammation index (SII) summarize acute-phase response, inflammatory-nutritional balance, and cellular immune activation. Cytokines may add information because they characterize immune pathways not fully captured by CRP, albumin, or blood counts; recent systematic-review evidence describes serum and urinary biomarkers as promising but heterogeneous and not yet clinically actionable in pediatric IgAV nephritis (8). A serum cytokine profiling study in children with established IgAV nephritis supports the feasibility of measurable circulating immune signatures (9), while pediatric acute-phase data implicate IL-17A, IL-18, and IL-23 in IgAV inflammation (10). Early clinical work also suggests that SII and CAR may have prognostic value in pediatric IgAV, but the evidence remains preliminary (11).

We therefore designed a single-center cohort to test whether a predefined Cytokine Augmentation Score (CAS), derived from interleukin (IL)-6, tumor necrosis factor alpha (TNF-α), IL-17A, IL-18, and IL-23, improves six-month incident nephritis risk stratification beyond clinical factors and a Routine Inflammatory Index Score (RIIS) based on CAR, NLR, and SII. The five cytokines were selected *a priori* because prior pediatric IgAV studies and mechanistic reviews supported complementary coverage of broad inflammatory/endothelial activation (IL-6 and TNF-α), the Th17 axis (IL-17A and IL-23), and inflammasome-associated signaling (IL-18) (6, 7, 9, 10). Grouping them as one module also avoided outcome-driven selection from the wider assay panel in this modest derivation cohort. We hypothesized that CAS would improve discrimination, calibration, Brier score, and net benefit for a surveillance-support decision.

## Methods

### Study design and participants

This single-center, prospective derivation cohort study was conducted at Wuhan Children's Hospital (Wuhan Maternal and Child Healthcare Hospital). The study screened children presenting from 1 January 2023 through 30 June 2024, with at least 6 months of follow-up. Reporting followed the Transparent Reporting of a multivariable prediction model for Individual Prognosis Or Diagnosis plus Artificial Intelligence (TRIPOD+AI) statement (12) and the Strengthening the Reporting of Observational Studies in Epidemiology (STROBE) guidance for cohort studies (13). The protocol conformed to the Declaration of Helsinki and was approved by the ethics committee of Wuhan Children's Hospital (Wuhan Maternal and Child Healthcare Hospital) (approval number: 2023R017-e03). All participants were younger than 18 years, and written informed consent to participate was obtained from a parent or legal guardian for every participant. The ethics committee of Wuhan Children's Hospital (Wuhan Maternal and Child Healthcare Hospital) approved this consent procedure.

The population comprised children younger than 18 years screened with clinically suspected IgAV during the study window. Time zero was the first hospital encounter for the current episode at which IgAV classification criteria were fulfilled and baseline laboratory testing was initiated. The baseline decision window was the first 24 h after time zero. To preserve temporal ordering, children who met nephritis criteria at any time during that baseline window were classified as having baseline nephritis and excluded; incident follow-up began after the baseline decision window. Inclusion required EULAR/PRINTO/PRES-classified IgAV, no nephritis during the baseline decision window, available baseline complete blood count with differential, platelet count, C-reactive protein (CRP), albumin, urinalysis, serum creatinine, and cytokine panel within the baseline window, and ascertainable six-month renal outcome. After exclusion of baseline renal involvement, IgAV classification required lower-limb predominant purpura or petechiae plus at least one nonrenal supportive feature, such as abdominal pain, arthritis or arthralgia, or IgA-predominant histopathology. Exclusion criteria were nephritis during the baseline decision window, alternative systemic inflammatory or autoimmune disease, known chronic kidney disease or primary IgA nephropathy, malignancy, immunodeficiency, inflammatory bowel disease, severe bacterial sepsis requiring intensive care, absence of any within-window cytokine result, and missing renal outcome data after retrieval attempts. A cytokine sample obtained after first in-hospital systemic steroid exposure but still within the baseline window was retained in the primary cohort, flagged as treatment-before-cytokine exposure, and removed in a predefined sensitivity analysis. Children with baseline nephritis were excluded from the primary analytic cohort to reduce reverse causation and ensure that model predictors preceded the incident renal endpoint.

### Predictors, outcomes, and follow-up

Baseline predictors were defined using the earliest available values in the baseline decision window, before the incident renal endpoint and before systemic glucocorticoid, intravenous immunoglobulin, or immunosuppressive treatment whenever a pretreatment value existed. CAR was CRP in mg/L divided by albumin in g/L; NLR was absolute neutrophil count divided by absolute lymphocyte count; and SII was platelet count multiplied by absolute neutrophil count divided by absolute lymphocyte count, with cell counts in 10^9^/L. RIIS was the mean of *z*-transformed natural-log CAR, natural-log NLR, and natural-log SII, thereby preserving the routine inflammatory signal while limiting collinearity. CRP values reported below the assay lower limit were replaced by the lower limit divided by the square root of 2 before CAR calculation and log transformation. The cytokine panel included IL-2, IL-6, TNF-α, IL-10, IL-17A, interferon gamma (IFN-γ), IL-18, IL-23, IL-12p70, and IL-4 in pg/mL. Serum cytokines were measured from the baseline sample by a multiplex bead-based flow-immunofluorescence assay in the hospital laboratory under routine internal quality-control procedures; samples were processed according to local laboratory standard operating procedures before analysis. No statistical batch correction was applied because the assay platform and laboratory quality-control procedures were consistent across the study period; assay lot was recorded where available, and laboratory personnel performing cytokine testing were blinded to clinical predictor/outcome data beyond information required to process the test and were not involved in endpoint adjudication. The five CAS components were selected *a priori* before outcome analysis from the 10-cytokine panel on the basis of prior pediatric IgAV evidence and complementary pathway coverage (9, 10). CAS was defined as the mean of *z*-transformed natural-log IL-6, natural-log TNF-α, natural-log IL-17A, natural-log IL-18, and natural-log IL-23. Values below the lower limit of quantification were set to the lower limit divided by the square root of 2, values above the upper reportable limit were set to the upper reportable value and flagged, and assay lot was recorded when available. RIIS and CAS were standardized using analytic-cohort means and standard deviations (SDs); these constants and assay-platform details are required for external implementation.

The primary outcome was incident IgAV nephritis within 6 months after the baseline decision window among children without baseline nephritis. Incident nephritis was defined by the first documented occurrence after the baseline decision window, confirmed by repeat testing or pediatric nephrology/rheumatology adjudication, of urine protein-to-creatinine ratio at least 0.2 mg/mg, dipstick protein at least 1+ in two samples, microscopic hematuria at least 5 red blood cells per high-power field or dipstick blood at least 1+ in two samples without another explanation, gross hematuria, hypertension with urinary abnormality, estimated glomerular filtration rate (eGFR) below 90 mL/min/1.73 m^2^ with urinary abnormality or adjudicated renal involvement, or more than 25% decline from baseline eGFR with urinary abnormality. Blood pressure was interpreted using pediatric percentile methods (14), and eGFR was calculated with the bedside Schwartz equation when height and creatinine were available (15). The six-month window allowed a 14-day grace period. Renal follow-up consisted of urinalysis and blood-pressure assessment at approximately 1, 2, 3, and 6 months after time zero, with additional testing when symptoms or clinician concern occurred. Confirmation required at least two abnormal urine tests separated by 1–14 days, except when gross hematuria, reduced eGFR with urinary abnormality, or specialist adjudication established nephritis. A single isolated episode of microscopic hematuria below confirmation criteria or trace proteinuria alone was not counted as the primary outcome. The primary logistic analysis included only children with six-month renal outcome ascertainment; censoring at last documented urine/blood-pressure assessment, transfer, withdrawal, or death applied only to the time-to-event sensitivity analysis. Clinically significant nephritis was defined as persistent proteinuria, gross hematuria, hypertension with urinary abnormality, or reduced eGFR with urinary abnormality, excluding isolated transient hematuria. Time to nephritis, clinically significant nephritis, persistent urinary abnormality at 12 months where available, and observed event rates by risk stratum were secondary outcomes.

### Model development, data quality, and statistical analysis

Because the objective was prognostic performance under observed care rather than causal treatment estimation, covariates were selected for availability before the outcome, clinical plausibility, and limited degrees of freedom. No data-driven univariable screening was used to select primary predictors; univariable logistic regressions were tabulated descriptively to check direction and scale but did not determine covariate inclusion. The primary comparator model included age, sex, symptom-to-blood-draw interval, gastrointestinal involvement, systolic blood-pressure percentile, subthreshold urinary warning flag, and RIIS; the augmented model added CAS. The subthreshold urinary warning flag was defined as trace dipstick protein or urinary red cells below the nephritis threshold during the baseline decision window. Post-baseline treatments, hospitalization duration, nephrology referral, and number of follow-up visits were not included as primary covariates. Consequently, the model estimates risk under local surveillance and treatment practice. Time-ordering bias was minimized by defining time zero and the baseline decision window before outcome accrual and excluding children with nephritis during that window.

No formal *a priori* sample-size calculation for prediction model development was performed; therefore, model complexity was prespecified to remain parsimonious. The achieved event-per-degree-of-freedom ratio was evaluated after cohort assembly as a descriptive stability check (16). The primary analysis required complete data for RIIS, CAS, the predefined clinical covariates, and the six-month renal outcome; missingness in secondary or descriptive variables was summarized by variable and outcome status when present. Variables with less than 20% missingness and clinical importance were eligible for multiple imputation by chained equations using at least 30 imputations in sensitivity or secondary analyses (17), whereas variables with more than 30% missingness were not eligible for adjusted modeling. Data-quality checks included duplicate detection, unit harmonization, physiologic range checks, verification of cytokine reportability flags, adjudication of uncertain renal outcomes by two reviewers with specialist resolution, and locking of the analysis dataset before final modeling.

Continuous variables were summarized as mean and standard deviation (SD) or median with interquartile range (IQR), and categorical variables as *n* and percentage. Standardized mean differences were emphasized for baseline comparisons, with *p* values reported descriptively. The primary analysis used multivariable logistic regression for six-month incident nephritis. Model 1 contained clinical variables and RIIS, and Model 2 added CAS. Incremental performance was assessed using the area under the receiver operating characteristic curve (AUC), Brier score, calibration intercept, calibration slope, likelihood-ratio test, and decision-curve net benefit. Decision-curve analysis evaluated the net benefit of using predicted risk to intensify urine and blood-pressure surveillance, not to initiate treatment; threshold probabilities from 0.05 to 0.50 were interpreted as surveillance-decision preferences and default strategies were no intensified surveillance and intensified surveillance for all (18). Exploratory risk strata were prespecified as low (<15%), intermediate (15% to <30%), and high (≥30%) predicted 6-month risk, based on clinically interpretable surveillance-intensity thresholds rather than data-derived cut points or treatment thresholds. Correlated AUCs were compared using a nonparametric approach (19). Internal validation used 1,000 bootstrap resamples to estimate optimism-corrected AUC, Brier score, and calibration slope. RIIS and CAS were modeled linearly per SD in the primary model because the event count was modest and the clinical goal favored an interpretable score. Restricted cubic splines were not used for primary inference.

Sensitivity and subgroup analyses: Sensitivity analyses included exclusion of children whose baseline cytokine draw occurred after first systemic steroid exposure, use of clinically significant nephritis as a stricter endpoint, Cox regression when event dates were reliable, replacement of RIIS with natural-log CAR, natural-log NLR, or natural-log SII one at a time, and complete-case analysis compared with imputed analysis where applicable. *Post hoc* score-construction checks compared equal-weighted CAS with a first-principal-component CAS and a variance-weighted CAS. Collinearity was examined with variance-inflation factors (VIFs), and the relationship between RIIS and CAS was summarized with Spearman correlations. Individual cytokines were evaluated only as exploratory markers, each entered one at a time in place of CAS and adjusted for the same clinical covariates plus RIIS, with Benjamini-Hochberg false-discovery rate (FDR) control (20). Limited predefined interaction analyses examined age group, sex, and gastrointestinal involvement. The renal endpoint and monitoring interpretation were cross-checked against contemporary kidney guidance (21).

Analyses were performed with R (Version 4.4.2) using the rms, pROC, mice, survival, car, and rmda packages.

## Results

### Baseline clinical characteristics

**Figure 1:**
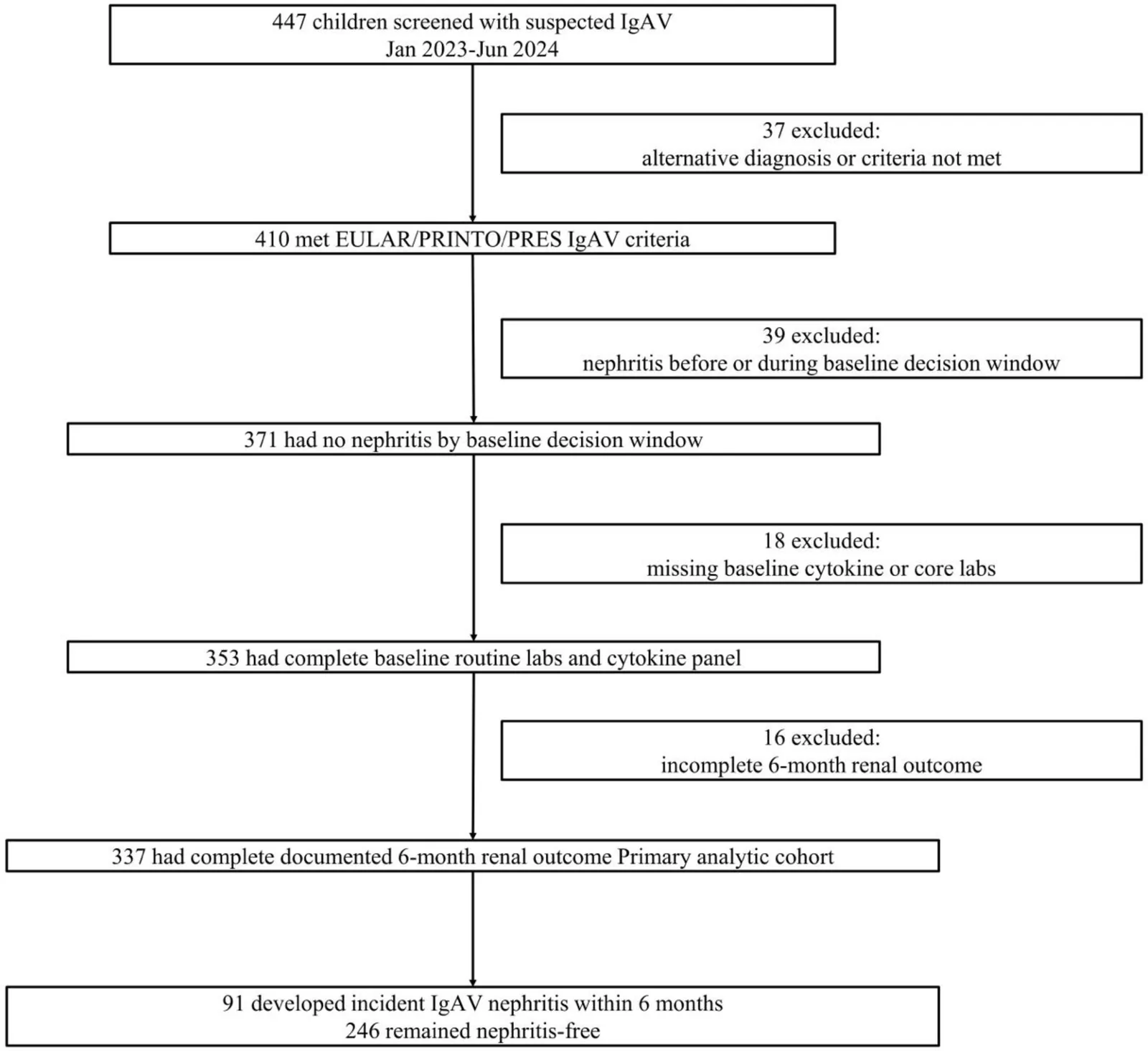
Participant flow from suspected IgA vasculitis screening to the six-month incident nephritis analytic cohort. Of 447 children screened, 410 met EULAR/PRINTO/PRES classification criteria, 371 had no nephritis during the 24 h baseline decision window, 353 had complete baseline routine and cytokine data, and 337 had six-month renal outcome ascertainment; 91 developed incident nephritis and 246 remained nephritis-free. EULAR, European League Against Rheumatism; IgAV, IgA vasculitis; PRES, Pediatric Rheumatology European Society; PRINTO, Pediatric Rheumatology International Trials Organization.

Of 447 screened children with suspected IgAV, 410 met EULAR/PRINTO/PRES classification criteria, 371 had no nephritis during the baseline decision window, 353 had complete baseline routine and cytokine data, and 337 had six-month renal outcome ascertainment (Figure 1). During follow-up, 91 children developed incident nephritis and 246 remained nephritis-free, giving a primary endpoint frequency of 27.0%. Clinically significant nephritis occurred in 47 children (13.9%) (Figure 1). With 91 incident nephritis events and 8 predictor degrees of freedom in the augmented model, the achieved event-per-degree-of-freedom ratio was 11.4.

The cohort had a mean age of 7.3 years, and 197 children (58.5%) were male (Table 1). Children who developed nephritis were older than those who remained nephritis-free (7.9 vs. 7.1 years; *p* = 0.026), whereas sex distribution was similar (61.5% vs. 57.3% male; *p* = 0.485). Joint involvement was more frequent in the nephritis group (59.3% vs. 45.9%; *p* = 0.029), and organ involvement count was higher (median 3 vs. 2; *p* = 0.001). Children with incident nephritis also had higher systolic blood-pressure percentile (69 vs. 65; *p* = 0.007), more frequent subthreshold urinary warning signs (42.9% vs. 18.7%; *p* < 0.001), higher serum IgA (1.99 vs. 1.63 g/L; *p* < 0.001), and higher D-dimer and fibrinogen.

**Table 1:** Baseline demographic and clinical characteristics according to six-month incident nephritis status in 337 children with IgA vasculitis.

Characteristic	Overall (*n* = 337)	No incident nephritis (*n* = 246)	Incident nephritis (*n* = 91)	SMD	*P* value
Age, years	7.3 (2.4)	7.1 (2.3)	7.9 (2.7)	0.30	0.026
Male sex, *n* (%)	197 (58.5)	141 (57.3)	56 (61.5)	0.09	0.485
BMI *z*-score	−0.2 (1.0)	−0.2 (1.0)	−0.3 (1.0)	−0.09	0.437
Symptom onset to baseline blood draw, days	3.8 [2.6, 5.9]	3.8 [2.5, 5.9]	3.9 [2.6, 5.6]	0.05	0.664
Inpatient index encounter, *n* (%)	208 (61.7)	147 (59.8)	61 (67.0)	0.15	0.222
Gastrointestinal involvement, *n* (%)	170 (50.4)	119 (48.4)	51 (56.0)	0.15	0.211
Joint involvement, *n* (%)	167 (49.6)	113 (45.9)	54 (59.3)	0.27	0.029
Organ involvement count	2 [2, 3]	2 [2, 3]	3 [2, 3]	0.41	0.001
Systolic BP percentile	67 [52, 77]	65 [52, 75]	69 [59, 84]	0.36	0.007
Diastolic BP percentile	58 [48, 70]	58 [48, 70]	60 [51, 72]	0.20	0.203
Subthreshold urinary warning flag, *n* (%)	85 (25.2)	46 (18.7)	39 (42.9)	0.52	<0.001
Serum creatinine, µmol/L	43.1 (7.7)	42.7 (7.4)	44.1 (8.5)	0.18	0.172
eGFR, mL/min/1.73 m^2^	117.0 (4.4)	117.2 (4.2)	116.5 (4.9)	−0.16	0.236
Serum IgA, g/L	1.72 [1.40, 2.14]	1.63 [1.35, 2.02]	1.99 [1.63, 2.29]	0.64	<0.001
C3, g/L	1.15 (0.20)	1.16 (0.20)	1.15 (0.20)	−0.05	0.699
D-dimer, mg/L FEU	0.56 [0.33, 0.83]	0.51 [0.30, 0.76]	0.70 [0.44, 0.98]	0.42	<0.001
Fibrinogen, g/L	3.1 (0.7)	3.0 (0.7)	3.3 (0.6)	0.42	<0.001

Values are mean (SD), median [IQR], or *n* (%). BMI, body mass index; BP, blood pressure; C3, complement component 3; eGFR, estimated glomerular filtration rate; FEU, fibrinogen-equivalent units; IgA, immunoglobulin A; IQR, interquartile range; *n*, number of participants; SD, standard deviation; SMD, standardized mean difference.

### Routine inflammatory indices and cytokine profile

Children with incident nephritis had higher CRP (13.9 vs. 9.3 mg/L; *p* < 0.001), lower albumin (38.9 vs. 40.4 g/L; *p* < 0.001), higher CAR (0.35 vs. 0.22; *p* < 0.001), higher NLR (3.00 vs. 2.21; *p* < 0.001), and higher SII (851 vs. 570; *p* < 0.001) (Table 2). The composite RIIS was also higher (0.28 vs. −0.14; *p* < 0.001). Descriptive cytokine comparisons showed significant differences after false-discovery-rate adjustment for all cytokines except IL-4, with prominent separation for IL-6, TNF-α, IL-17A, IL-18, and IL-23. CAS was also higher among children who developed nephritis (0.65 vs. −0.23; *p* < 0.001).

**Table 2:** Baseline routine inflammatory indices, circulating cytokine concentrations, and composite scores according to six-month incident nephritis status.

Marker	Overall (*n* = 337)	No incident nephritis (*n* = 246)	Incident nephritis (*n* = 91)	*P* value	FDR q*
CRP, mg/L	10.4 [5.9, 17.0]	9.3 [5.2, 15.3]	13.9 [8.8, 28.4]	<0.001	—
Albumin, g/L	39.9 [37.9, 42.2]	40.4 [38.4, 42.6]	38.9 [37.0, 40.5]	<0.001	—
CAR	0.26 [0.14, 0.43]	0.22 [0.13, 0.39]	0.35 [0.22, 0.77]	<0.001	—
NLR	2.41 [1.46, 4.15]	2.21 [1.31, 3.73]	3.00 [1.89, 5.64]	<0.001	—
SII	640 [345, 1,072]	570 [313, 983]	851 [525, 1,606]	<0.001	—
RIIS	0.02 [−0.61, 0.62]	−0.14 [−0.76, 0.48]	0.28 [−0.23, 1.17]	<0.001	—
IL-2, pg/mL	3.96 [2.65, 6.02]	3.75 [2.46, 5.48]	4.92 [3.23, 7.20]	<0.001	<0.001
IL-6, pg/mL	6.68 [3.80, 12.23]	5.58 [3.11, 10.13]	11.83 [6.59, 23.01]	<0.001	<0.001
TNF-*α*, pg/mL	12.99 [7.41, 22.37]	10.96 [6.22, 18.47]	21.14 [12.22, 41.10]	<0.001	<0.001
IL-10, pg/mL	7.50 [5.16, 11.41]	7.12 [4.73, 10.32]	9.94 [6.38, 13.33]	<0.001	<0.001
IL-17A, pg/mL	8.64 [4.94, 15.67]	7.16 [4.23, 12.13]	15.67 [8.83, 24.52]	<0.001	<0.001
IFN-γ, pg/mL	9.69 [6.26, 14.93]	8.78 [5.35, 13.37]	12.25 [8.28, 21.16]	<0.001	<0.001
IL-18, pg/mL	213 [120, 400]	171 [102, 303]	411 [222, 695]	<0.001	<0.001
IL-23, pg/mL	35.0 [19.4, 60.0]	28.2 [16.7, 46.4]	59.8 [34.6, 97.2]	<0.001	<0.001
IL-12p70, pg/mL	2.99 [2.08, 4.53]	2.91 [2.03, 4.26]	3.33 [2.41, 5.43]	0.006	0.007
IL-4, pg/mL	2.46 [1.78, 3.48]	2.37 [1.65, 3.50]	2.63 [2.05, 3.47]	0.122	0.122
CAS	−0.04 [−0.66, 0.60]	−0.23 [−0.85, 0.35]	0.65 [0.04, 1.23]	<0.001	—

CAR, RIIS, and CAS were calculated at the individual-patient level before summarization. Values are median [IQR] unless otherwise indicated. CAR, C-reactive protein-to-albumin ratio; CAS, cytokine augmentation score; CRP, C-reactive protein; FDR, false-discovery rate; IFN-γ, interferon gamma; IgA, immunoglobulin A; IL, interleukin; IQR, interquartile range; NLR, neutrophil-to-lymphocyte ratio; q, FDR-adjusted *p* value; RIIS, routine inflammatory index score; SII, systemic immune-inflammation index; TNF-α, tumor necrosis factor alpha.

*FDR *q*-values apply to exploratory cytokine comparisons only; routine inflammatory markers and composite scores were not included in the cytokine FDR family.

### Primary model and incremental performance

**Figure 2:**
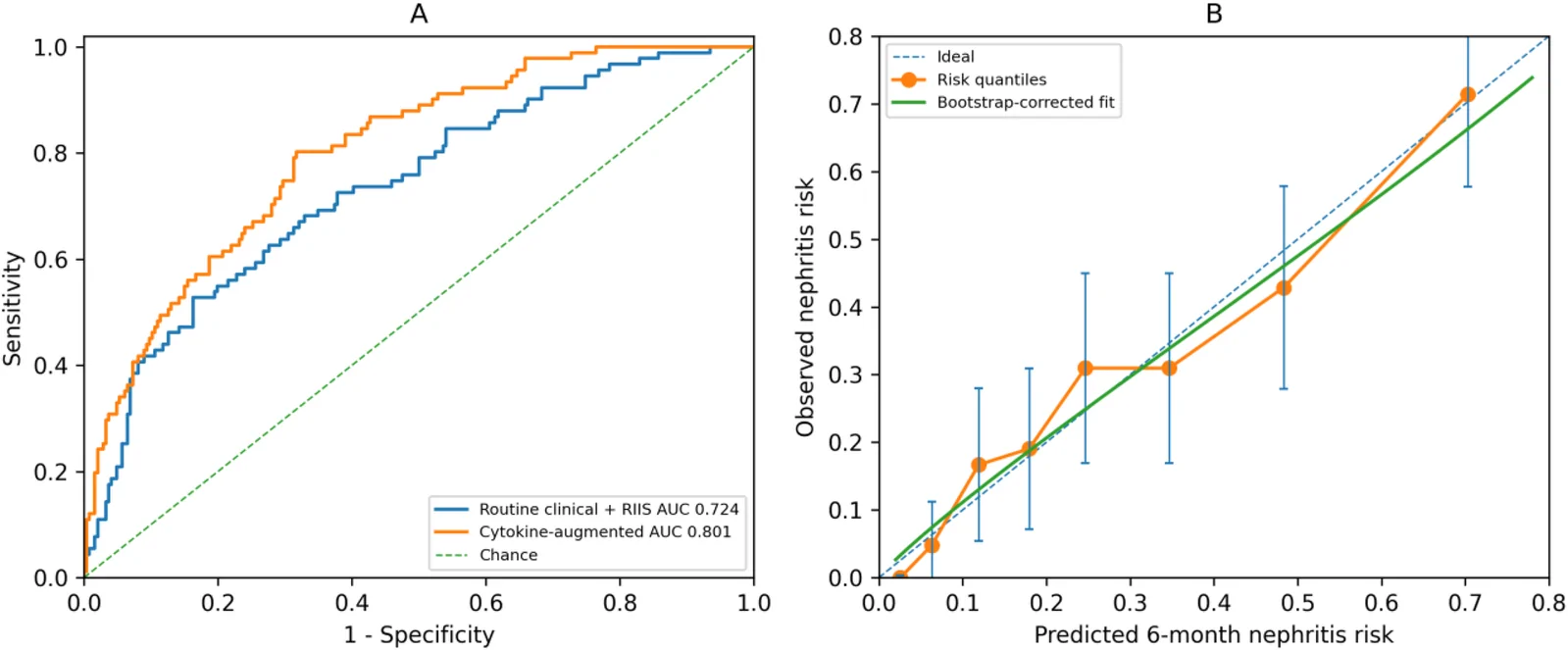
Receiver operating characteristic curves and internal calibration for six-month incident nephritis prediction. **(A)** ROC curves compare the routine clinical plus RIIS model (AUC, 0.724) with the cytokine-augmented model (AUC, 0.801); the diagonal line represents chance discrimination. **(B)** The augmented-model calibration plot shows observed vs. predicted six-month nephritis risk across grouped risk quantiles, with 95% confidence intervals, the ideal line, and the bootstrap-corrected calibration fit. AUC, area under the receiver operating characteristic curve; RIIS, routine inflammatory index score; ROC, receiver operating characteristic.

**Figure 3:**
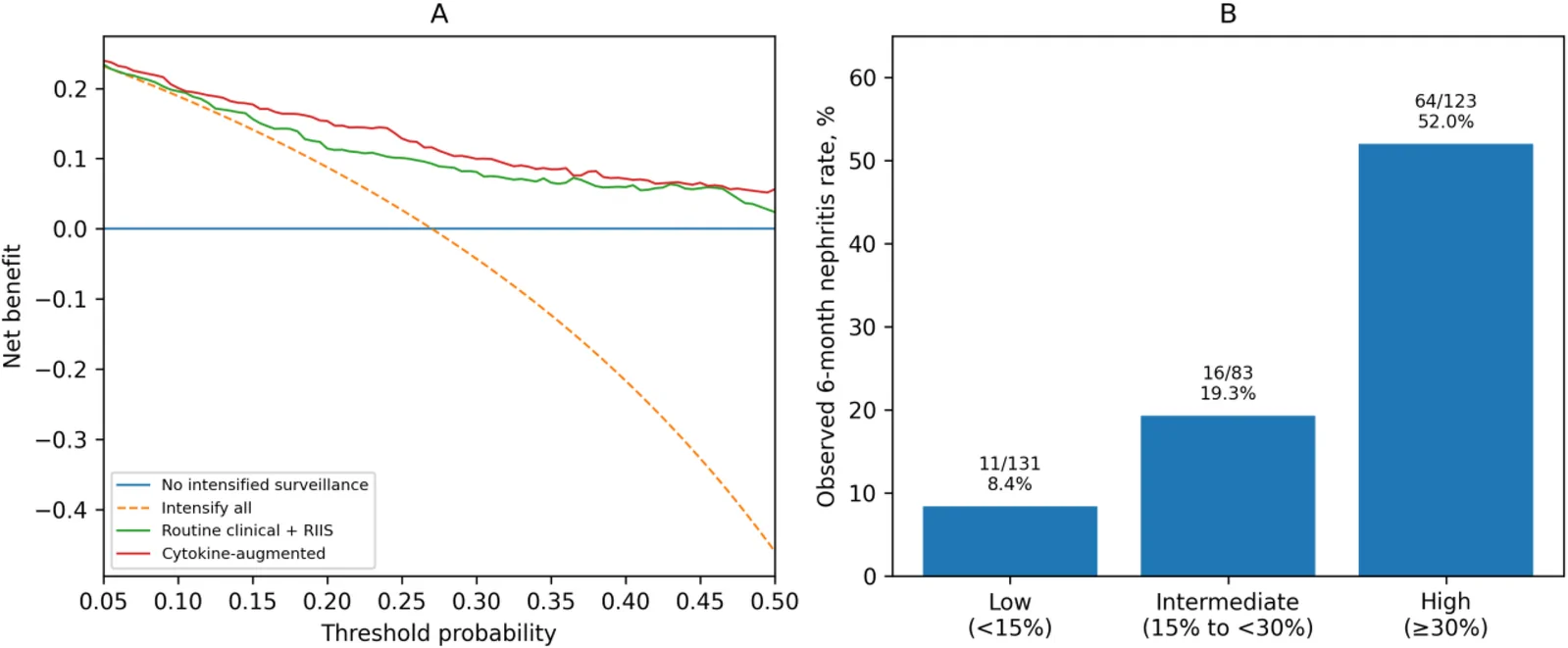
Decision-curve analysis for intensified renal surveillance and observed six-month nephritis rates across predefined risk strata. **(A)** Net benefit is shown from threshold probabilities of 0.05–0.50 for no intensified surveillance, intensified surveillance for all the routine clinical plus RIIS model, and the cytokine-augmented model., and **(B)** Observed nephritis rates were 8.4% (11/131), 19.3% (16/83), and 52.0% (64/123) in the low (<15%), intermediate (15% to <30%), and high (≥30%) predicted-risk strata, respectively. CAS, cytokine augmentation score; RIIS, routine inflammatory index score.

CAS was independently associated with six-month incident nephritis (adjusted odds ratio (OR) per 1-SD increase, 2.97; 95% CI, 2.01–4.39; *p* < 0.001), whereas RIIS was not independently associated after CAS entered the model (OR, 1.10; 95% CI, 0.77–1.56; *p* = 0.609) (Table 3). The full augmented-model equation was: logit (*p*) = −2.42 + 0.131 ×  age + 0.399 × male − 0.031 × symptom-to-blood-draw interval + 0.278 × gastrointestinal involvement −0.010 × (systolic BP percentile/10) + 1.102 × subthreshold urinary warning flag + 0.095 × RIIS + 1.089 × CAS, where binary variables were coded 1 = yes and RIIS/CAS were standardized scores. The baseline subthreshold urinary warning flag also remained associated with nephritis (OR, 3.01; 95% CI, 1.65–5.48; *p* < 0.001). Adding CAS increased the apparent AUC from 0.724 (95% CI, 0.663–0.780) to 0.801 (95% CI, 0.752–0.852), with a difference in AUC of 0.077 (95% CI, 0.035–0.124; *p* = 0.001) (Figure 2). Brier score improved from 0.168 to 0.149, and the likelihood-ratio test for adding CAS was significant (*χ*^2^(1) = 35.84; *p* < 0.001). Bootstrap internal validation yielded an optimism-corrected AUC of 0.782, a Brier score of 0.159, and a calibration slope of 0.90. Decision-curve analysis for an intensified-surveillance action favored the augmented model across the evaluated threshold range, and exploratory risk strata separated observed nephritis rates: 11/131 (8.4%), 16/83 (19.3%), and 64/123 (52.0%) for low-, intermediate-, and high-risk groups, respectively (Figure 3). These stratum-specific rates were crude observed six-month proportions under local follow-up and treatment practice; they were not competing-risk-adjusted because early immunosuppressive therapy was considered a post-baseline management exposure rather than a renal-event-precluding outcome.

**Table 3:** Multivariable cytokine-augmented logistic regression model and incremental performance for predicting six-month incident nephritis.

(A). Multivariable cytokine-augmented logistic regression model		
Predictor in augmented model	Adjusted OR (95% CI)	*P* value
Age, per 1 year	1.14 (1.01, 1.29)	0.034
Male sex	1.49 (0.82, 2.69)	0.188
Symptom-to-blood-draw interval, per day	0.97 (0.87, 1.08)	0.552
Gastrointestinal involvement	1.32 (0.75, 2.32)	0.332
Systolic BP percentile, per 10-point increase	0.99 (0.82, 1.18)	0.884
Subthreshold urinary warning flag	3.01 (1.65, 5.48)	<0.001
RIIS, per 1-SD increase	1.10 (0.77, 1.56)	0.609
CAS, per 1-SD increase	2.97 (2.01, 4.39)	<0.001

(B). Incremental performance after adding CAS to the routine clinical plus RIIS model			
Metric	Routine clinical + RIIS model	Cytokine-augmented model	Incremental result
AUC	0.724 (0.663, 0.780)	0.801 (0.752, 0.852)	Δ0.077 (0.035, 0.124); *p* = 0.001
Brier score	0.168	0.149	Absolute reduction 0.019
Apparent calibration intercept/slope	—	0.00/1.00	Optimism-corrected slope 0.90
Bootstrap optimism-corrected AUC/Brier	—	0.782/0.159	Internal validation only
Likelihood-ratio test for adding CAS	Reference	Augmented	*χ*^2^ (1) = 35.84; *p* < 0.001

AUC, area under the receiver operating characteristic curve; BP, blood pressure; CAS, cytokine augmentation score; CI, confidence interval; IgA, immunoglobulin A; OR, odds ratio; RIIS, routine inflammatory index score; SD, standard deviation. Binary predictors were coded yes = 1/no = 0; age, symptom-to-blood-draw interval, systolic BP percentile, RIIS, and CAS were modeled as specified in the predictor column. Calibration intercept/slope are apparent for the augmented model unless otherwise specified.

### Sensitivity and exploratory analyses

Excluding 24 children whose baseline cytokine draw occurred after systemic steroid exposure left 313 children and 83 events; the rounded CAS estimate was similar (OR, 2.97; 95% CI, 1.98–4.46; *p* < 0.001) (Table 4). Using clinically significant nephritis as a stricter endpoint yielded a similar effect (OR, 3.01; 95% CI, 1.85–4.89; *p* < 0.001), and the time-to-event Cox sensitivity model gave a hazard ratio (HR) of 2.00 (95% CI, 1.55–2.57; *p* < 0.001). Replacing RIIS with natural-log CAR, natural-log NLR, or natural-log SII did not materially change the CAS estimate. Interactions by age group, sex, and gastrointestinal involvement were not significant, so no subgroup-specific claim was made. Exploratory individual cytokine analyses identified IL-18, IL-6, IL-23, TNF-α, and IL-17A after FDR control, supporting the predefined CAS module. Exploratory individual-cytokine models had AUCs of 0.781 for IL-18, 0.773 for IL-6, 0.770 for IL-23, 0.759 for TNF-α, and 0.764 for IL-17A, all below the equal-weight CAS AUC of 0.801. The RIIS–CAS Spearman correlation was moderate (*ρ* = 0.43), and all component VIFs were below 3.2; alternative first-principal-component and variance-weighted CAS constructions yielded similar AUCs of 0.798 and 0.800, respectively (Table 5). Overall, robustness checks supported the primary incremental-value result while preserving the need for external validation.

**Table 4:** Sensitivity, interaction, and exploratory cytokine analyses for six-month incident nephritis.

Analysis	Participants/events	Effect estimate	*P* value	Model AUC
Primary augmented model	337/91	CAS OR 2.97 (2.01, 4.39)	<0.001	0.801
Exclude baseline cytokine draws after systemic steroid exposure	313/83	CAS OR 2.97 (1.98, 4.46)	<0.001	0.799
Clinically significant nephritis endpoint	337/47	CAS OR 3.01 (1.85, 4.89)	<0.001	0.824
Time-to-event Cox sensitivity model	337/91	CAS HR 2.00 (1.55, 2.57)	<0.001	—
Replace RIIS with log CAR	337/91	CAS OR 2.86 (1.94, 4.23)	<0.001	0.799
Replace RIIS with log NLR	337/91	CAS OR 3.04 (2.08, 4.45)	<0.001	0.803
Replace RIIS with log SII	337/91	CAS OR 3.04 (2.05, 4.50)	<0.001	0.802
Interaction: CAS × age ≥8 years	337/91	Interaction term	0.507	—
Interaction: CAS × male sex	337/91	Interaction term	0.351	—
Interaction: CAS × GI involvement	337/91	Interaction term	0.104	—
Exploratory individual cytokine: IL-18	337/91	OR 2.89 (1.99, 4.20); FDR *q* < 0.001	<0.001	0.781
Exploratory individual cytokine: IL-6	337/91	OR 2.72 (1.88, 3.93); FDR *q* < 0.001	<0.001	0.773
Exploratory individual cytokine: IL-23	337/91	OR 2.68 (1.87, 3.83); FDR *q* < 0.001	<0.001	0.770
Exploratory individual cytokine: TNF-*α*	337/91	OR 2.55 (1.78, 3.66); FDR *q* < 0.001	<0.001	0.759
Exploratory individual cytokine: IL-17A	337/91	OR 2.58 (1.78, 3.73); FDR *q* < 0.001	<0.001	0.764
Alternative CAS construction: first principal component score	337/91	CAS-PC1 OR 2.93 (1.99, 4.32)	<0.001	0.798
Alternative CAS construction: variance-weighted score	337/91	Weighted CAS OR 2.95 (2.00, 4.35)	<0.001	0.800

Values are effect estimates per 1-SD score increase unless otherwise specified. AUC, area under the receiver operating characteristic curve; CAS, cytokine augmentation score; CAR, C-reactive protein-to-albumin ratio; CI, confidence interval; FDR, false-discovery rate; GI, gastrointestinal; HR, hazard ratio; IgA, immunoglobulin A; IL, interleukin; NLR, neutrophil-to-lymphocyte ratio; OR, odds ratio; PC1, first principal component; RIIS, routine inflammatory index score; SD, standard deviation; SII, systemic immune-inflammation index; TNF-α, tumor necrosis factor alpha. Individual cytokine analyses were exploratory, used one cytokine at a time in place of CAS, and were adjusted for the same clinical covariates plus RIIS with Benjamini-Hochberg FDR control. Alternative score-construction analyses were *post hoc* sensitivity checks.

**Table 5:** Relationships, collinearity, and alternative construction diagnostics for RIIS and CAS.

Variable or diagnostic	Spearman *ρ* with RIIS	Spearman *ρ* with CAS	VIF or sensitivity result
RIIS	1.00	0.43	—
CAS	0.43	1.00	—
Natural-log CAR	0.71	0.28	1.44
Natural-log NLR	0.83	0.31	3.18
Natural-log SII	0.86	0.33	3.09
Natural-log IL-6	0.32	0.76	1.66
Natural-log TNF-α	0.30	0.72	1.59
Natural-log IL-17A	0.29	0.75	1.74
Natural-log IL-18	0.36	0.81	1.51
Natural-log IL-23	0.33	0.78	1.62
First-principal-component CAS	0.41	0.96	AUC 0.798
Variance-weighted CAS	0.42	0.98	AUC 0.800

AUC, area under the receiver operating characteristic curve; CAR, C-reactive protein-to-albumin ratio; CAS, cytokine augmentation score; IgA, immunoglobulin A; IL, interleukin; NLR, neutrophil-to-lymphocyte ratio; ρ, Spearman correlation coefficient; RIIS, routine inflammatory index score; SII, systemic immune-inflammation index; TNF-α, tumor necrosis factor alpha; VIF, variance-inflation factor. VIFs were calculated within the corresponding component set; all values were below the conventional threshold suggesting problematic collinearity. Alternative CAS constructions were *post hoc* sensitivity analyses.

## Discussion

This single-center pediatric IgAV cohort found that a predefined cytokine augmentation score was associated with improved internally evaluated six-month incident nephritis risk stratification beyond routine clinical variables and a CAR/NLR/SII-based inflammatory score. The principal result is promising but should be interpreted as internal derivation evidence. The event-per-degree-of-freedom ratio of 11.4 and absence of a formal Riley sample-size calculation mean coefficient stability should be considered exploratory despite bootstrap validation (16). The observed risk strata were clinically interpretable in the derivation cohort, with nephritis rates of 8.4%, 19.3%, and 52.0% across low-, intermediate-, and high-risk groups.

CAS combined IL-6 and TNF-α, which represent broad inflammatory and endothelial-activation signals, IL-23 and IL-17A, which are consistent with a Th17-related axis, and IL-18, which can reflect inflammasome-linked inflammatory activation (6, 7, 9, 10). Equal weighting was used for parsimony and to avoid overfitting, not because the five mediators are assumed to be biologically equivalent. *Post hoc* principal component analysis (PCA) and variance-weighted versions were strongly concordant with the equal-weighted score and did not improve AUC, supporting retention of the simpler prespecified score for this derivation analysis. This pathway interpretation is hypothesis-generating because the study measured circulating proteins and cannot localize kidney tissue mechanisms. The exploratory finding that IL-18, IL-6, IL-23, TNF-α, and IL-17A each showed associations after FDR control supports the internal coherence of the score, but it does not prove that any single cytokine causes nephritis. The design is prognostic and cytokines may represent a systemic inflammatory state that precedes clinically detectable renal findings. The moderate RIIS–CAS correlation and attenuation of RIIS after CAS entered the model suggest partially overlapping inflammatory information plus incremental cytokine signal; this prognostic analysis should not be interpreted as evidence that cytokines causally mediate the association between routine inflammatory markers and nephritis. This distinction matters because the goal is not to substitute a cytokine panel for clinical assessment; rather, it is to determine whether immune information available at the index encounter improves the targeting of renal surveillance.

Existing pediatric prediction studies have demonstrated that renal risk prediction is feasible but often limited by risk of bias, inconsistent reporting, and inadequate validation (22). Recent biomarker studies also emphasize that statistically associated serum and urinary biomarkers are not yet clinically actionable without prospective validation and standardization (8). The cytokine component is consistent with reports of altered serum cytokine profiles and IL-17A/IL-18/IL-23 elevations in pediatric IgAV, while the present study tested whether that information added value to routine clinical variables and inflammatory markers (9, 10). This study addresses several methodological concerns by excluding children with nephritis during the baseline decision window, using a predefined cytokine module instead of unrestricted cytokine selection, reporting calibration and Brier score, and anchoring decision-curve analysis to intensified surveillance. The observed importance of baseline urinary warning signs is consistent with clinical expectation, while the persistence of CAS after adjustment suggests that cytokine information is not merely a surrogate for trace urinary abnormalities. Compared with studies focused on established nephritis, this incident-outcome design is more directly relevant to the common clinical scenario in which a child has IgAV but no definite nephritis at presentation.

Children in the high-risk stratum had an observed nephritis rate of 52.0%. After external validation, such a result could help identify children for whom more intensive early urine and blood-pressure follow-up, a lower threshold for pediatric nephrology review, and more explicit family counseling about delayed renal findings may be considered. Children in the low-risk stratum still had an 8.4% event rate; therefore, the model should not be used to discontinue guideline-concordant monitoring. Its most realistic use is to prioritize appointment timing, reinforce adherence, and identify children for prospective multicenter validation. Decision-curve results are interpretable only for the specified action of intensified surveillance, not for treatment initiation. In practice, a high-risk result would be useful only if it changes the reliability or timing of follow-up and if external validation confirms transportability.

This is a single-center Chinese cohort, so prediction performance may not transport to other centers, assay platforms, or follow-up systems. Cytokine testing may be more available in tertiary pediatric centers than in lower-resource settings, and CAS values may vary with assay platform, lot, sample handling, storage, and timing relative to systemic steroids. External reproducibility requires detailed assay-platform reporting, including manufacturer, quantification limits, coefficients of variation, batch/lot distribution, censored-value counts, and technician blinding. Practical implementation would also require reliable sample-processing timelines, inter-platform calibration, local reference distributions, reimbursement or institutional support, and clear workflows linking a high-risk result to surveillance rather than treatment. Residual selection bias may persist if children with complete cytokine panels differ systematically from those without such testing. Outcome ascertainment may also be influenced by follow-up intensity, because children with greater baseline concern may have more urine testing opportunities. Some renal outcomes may be missed if follow-up urinalysis occurred outside the hospital network, although active retrieval and sensitivity analyses were planned. Baseline renal biopsy/pathology and renal imaging were not systematically available because participants had no nephritis at baseline and such procedures were not clinically indicated; therefore, subclinical renal pathology or imaging abnormalities could not be evaluated. The 52.0% high-risk incidence was not competing-risk-adjusted; early immunosuppressive therapy may modify observed renal risk and should be addressed in future time-to-event or causal analyses. The model lacks external validation and should not be described as validated or clinically deployed until tested in independent centers (23).

In conclusion, a predefined cytokine augmentation score was associated with improved internally evaluated six-month incident nephritis risk stratification beyond clinical variables and routine CAR/NLR/SII-based assessment in children with IgAV who had no nephritis during the baseline decision window. The model is best interpreted as an investigational tool for supporting surveillance intensity, not as a replacement for guideline-concordant monitoring. External multicenter validation, complete model-equation reporting, assay standardization, and practical cost-effectiveness assessment are required before clinical implementation.

## Data Availability

The raw data supporting the conclusions of this article will be made available by the authors, without undue reservation.
